# Academy of Medicine Notes

**Published:** 1893-05

**Authors:** 


### Academy of Medicine Notes.

THE next stated meeting of the Academy will be held June 20th, under the auspices of the section on Obstetrics and Gynecology.

AT THE last meeting of the Section on Obstetrics and Gynecology, the following amendment was offered to the constitution: "The election of officers shall occur at the regular meeting in May."

The names of all members of the old societies, now comprising the Academy, who shall not have paid their initiation fee by June 21st, will be dropped from the Secretary's rolls. They can then only be admitted to the Academy through the Council.

---

A committee, composed of Drs. Hayd, Krauss, and Dagenais, was appointed by the Council to oversee the furnishing of the new rooms. Fellows of the Academy desiring to donate suitable engravings, books, or periodicals, will please confer with this committee.

---

It is to be hoped that when the Academy is settled in its new home, its membership will be greatly increased. Physicians residing at a short distance from Buffalo should avail themselves of the benefits of the Academy, and spend their Tuesday evenings pleasantly and profitably with the progressive members of the Buffalo profession. The initiation fee for non-resident fellows is $3.00, the annual dues $5.00.

---

The section on Surgery will meet in the new home of the Academy at its next regular meeting. This section will meet twice during the month of May—May 2d and May 30th. The Section on Medicine will meet June 6th; Section on Pathology, June 13th; and the Section on Obstetrics, June 20th. This change was necessitated by the clause in the constitution stating that the annual meeting of the Academy shall be held one week after the stated meeting in June; consequently, the annual meeting will be held June 27th.

---

The programme for the next meeting of the Section on Surgery will be on the "Symptomatology and Medical Treatment of Intestinal Obstructions," by Dr. Henry R. Hopkins. Surgical Treatment, by Dr. Roswell Park. Discussion, by Dr. Matthew D. Mann and Dr. Charles G. Stockton. For the May 30th meeting of this section, the subject will be on "Hemorrhoids," by Dr. Edward G. Meyer; "Treatment of Hemorrhoids," by Dr. Edward Clark. This will be the last meeting of this Section until September 5th.

